# Retraction Note: IL-1RA inhibits esophageal carcinogenesis and lymphangiogenesis via downregulating VEGF-C and MMP9

**DOI:** 10.1007/s10142-024-01368-1

**Published:** 2024-05-11

**Authors:** Zhimin Shen, Peipei Zhang, Weiguang Zhang, Fei Luo, Hui Xu, Shuchen Chen, Mingqiang Kang

**Affiliations:** 1 Department of Thoracic Surgery, Fujian Medical University, Union Hospital, 350001 Fuzhou, China; 2 Key Laboratory of Gastrointestinal Cancer(Fujian Medical University), Ministry of Education, 350122 Fuzhou, China; 3 Fujian Key Laboratory of Tumor Microbiology, Fujian Medical University, Fuzhou, 350122 Fujian, China; 4 Key Laboratory of Cardio‑Thoracic Surgery, (Fujian Medical University), Fujian Province University, Fuzhou, China

**Functional & Integrative Genomics (2023) 23:164**

10.1007/s10142-023-01049-5

The Editor-in-Chief and the publisher have retracted this article. The article was submitted to be part of a guest-edited issue. An investigation by the publisher found a number of articles, including this one, with a number of concerns, including but not limited to compromised editorial handling and peer review process, inappropriate or irrelevant references or not being in scope of the journal or guest-edited issue. Based on the investigation’s findings, the Editor-in-Chief therefore no longer has confidence in the results and conclusions of this article.

The author, Mingqiang Kang, has stated that all authors disagree with this retraction

